# Sharpin suppresses β1-integrin activation by complexing with the β1 tail and kindlin-1

**DOI:** 10.1186/s12964-019-0407-6

**Published:** 2019-08-20

**Authors:** Juan Gao, Yun Bao, Shushu Ge, Peisen Sun, Jiaojiao Sun, Jianmin Liu, Feng Chen, Li Han, Zhongyuan Cao, Jun Qin, Gilbert C. White, Zhen Xu, Yan-Qing Ma

**Affiliations:** 1 Collaborative Research Program for Cell Adhesion Molecules, Shanghai University School of Life Sciences, Shanghai, China; 2 Department of Molecular Cardiology, Lerner Research Institute Cleveland Clinic, Cleveland, OH USA; 3 Blood Research Institute, Versiti, 8727 Watertown Plank Road, Milwaukee, WI 53226 USA; 4 Department of Biochemistry, Medical College of Milwaukee, Milwaukee, WI USA

**Keywords:** Sharpin, Kindlin-1, Integrin, Talin

## Abstract

**Background:**

Previously sharpin has been identified as an endogenous inhibitor of β1-integrin activation by directly binding to a conserved region in the cytoplasmic tails (CTs) of the integrin β1-associated α subunits.

**Methods:**

Here we employed biochemical approaches and cellular analyses to evaluate the function and molecular mechanism of the sharpin-kindlin-1 complex in regulating β1-integrin activation.

**Results:**

In this study, we found that although the inhibition of sharpin on β1-integrin activation could be confirmed, sharpin had no apparent effect on integrin αIIbβ3 activation in CHO cell system. Notably, a direct interaction between sharpin and the integrin β1 CT was detected, while the interaction of sharpin with the integrin αIIb and the β3 CTs were substantially weaker. Importantly, sharpin was able to inhibit the talin head domain binding to the integrin β1 CT, which can mechanistically contribute to inhibiting β1-integrin activation. Interestingly, we also found that sharpin interacted with kindlin-1, and the interaction between sharpin and the integrin β1 CT was significantly enhanced when kindlin-1 was present. Consistently, we observed that instead of acting as an activator, kindlin-1 actually suppressed the talin head domain mediated β1-integrin activation, indicating that kindlin-1 may facilitate recruitment of sharpin to the integrin β1 CT.

**Conclusion:**

Taken together, our findings suggest that sharpin may complex with both kindlin-1 and the integrin β1 CT to restrict the talin head domain binding, thus inhibiting β1-integrin activation.

## Background

The interaction of cells and extracellular matrix (ECM) in metazoans is tightly regulated by cell adhesion molecules, especially the integrin family members [1]. The integrin-mediated crosstalk between cells and ECM is dynamically regulated by turnover of integrin activation. Dysfunction of integrin activation associates with multiple pathological conditions, such as inflammation, skin fragility, thrombosis and cancer [2, 3]. Integrin activation is a process of conformational changes from a resting state to an active state which allows integrins to bind their extracellular ligands. Integrin activation is precisely modulated, either positively or negatively, through the dialogue between the integrin α/β cytoplasmic tails (CTs) and their intracellular binding partners [4–6].

Among many integrin CT-binding partners, the talin head domain is an essential integrin activator that has been extensively studied [7–9]. At the resting state, the integrin α/β CTs interact with each other and form a membrane-proximal complex [10]. Upon stimulation, the talin head domain can be released and interacts with the conserved NPxY motif and some membrane-proximal residues in the integrin β CT, thus being able to unclasp the integrin α/β CT complex and trigger integrin activation [7, 11, 12]. The kindlin family (kindlin-1, − 2 and − 3) represents another class of the integrin CT-binding proteins [13–15]. Kindlin and the talin head domain can simultaneously bind to the integrin β CT and cooperatively support integrin activation [16, 17]. Kindlin binds to the second NxxY motif at the C-termini of integrin β CT, but kindlin itself has no capacity to unclasp the integrin α/β CT complex and thus fails to induce integrin activation [16, 18, 19]. Although kindlin has been identified as an important co-activator to support the talin head domain induced integrin activation [16, 19–21], a role of kindlin in suppressing β1-integrin activation has also been reported [18]. Since both the talin head domain and kindlin can simultaneously bind the integrin β CT and have no significant interaction between each other [17], we postulate that the role of the kindlin family members in regulating integrin activation may depend on their binding partners in cells.

Sharpin, a key component of the linear ubiquitin chain assembly complex (LUBAC), which consists of three subunits (HOIL-1, HOIP and sharpin), has been revealed to play critical roles in multiple cellular signaling pathways and pathological events [22]. Mice expressing loss-of-function sharpin exhibit multi-organ defects, including chronic inflammation and immunodeficiency [23–25], which are possibly ascribed to the dysfunction of NF-κB activation and apoptotic signaling pathways [22, 26–31]. Sharpin has been found to be highly expressed in many types of tumors [32–35], indicating that it may possess oncogene features. Interestingly, sharpin has been recently identified as a binding partner of the integrin α CT and can suppress both β1- and β2-integrin activation by blocking recruitment of talin and kindlin [32, 36, 37]. In addition, the role of sharpin in inhibiting integrin activation and regulating NF-κB signaling seem to be mutually exclusive [37].

Since the talin head domain and kindlin simultaneously bind to the integrin β CT without mutual exclusion [17], it is difficult to explain how sharpin, as an integrin α CT-binding protein, can spatially disrupt binding of both talin and kindlin to the integrin β CT [32]. In this study, by evaluating the regulation of sharpin on the activation of different integrins and testing its interaction with different integrin α/β CTs, we delineate a novel mechanism by which sharpin can specifically suppress integrin activation in an integrin β CT-specific manner.

## Methods

### Antibodies, plasmids and proteins

Flag antibody (SG4110–16, Shanghai Genomics), 6 × his antibody (SG4110–06, Shanghai Genomics), sharpin antibody (ab174545 and ab69507, Abcam), FAK antibody (#3283, CST) and Y-FAK antibodies (#3285, CST) were used for immunoblotting; PAC1 antibody (340535, BD Biosciences), 9EG7 antibody (553715, BD Biosciences) and 7E2 antibody (DSHB) were used for FACS analysis. Plasmid of GST-fibronectin type III repeats 9–11 (GST-Fn-III) was kindly provided by David Calderwood [18]. The cDNA of full length sharpin was kindly provide by Ivan Dikic [26], and subcloned into vectors of pET28a, pHis-1, pGST-1 and pGADT7 for different experiments. The CT of integrin α5β1 and integrin αIIbβ3 were subcloned into pGST-1 vector. Kindlin-1 was subcloned into pET31b, pGST-1 and pGBKT7 vectors. To express and purify proteins, the expression vectors were transformed into Rosetta DE3 strain and induced to express proteins with 0.4 mM of IPTG. GST-tagged or his-tagged proteins were purified by Glutathione Sepharpose (GE) and Ni-NTA agarose (Qiagen) respectively, according to the manufactures’ protocols. The purified GST-Fn-III protein was further labeled with biotin (EZ-Link™ NHS-Biotin, Thermo Fisher).

### Integrin activation assays

Integrin αIIbβ3 activation in CHO cells was measured by flow cytometry using PAC-1 antibody as previously described [38]. In brief, DsRed-fused talin head domain, EGFP-fused kindlin-1 and flag-fused sharpin were co-expressed by transfection in CHO cells that stably express integrin αIIbβ3 (CHO-αIIbβ3). 24 h after transfection, CHO-αIIbβ3 cells were harvested and incubated with PAC-1 (an antibody specific for activated integrin αIIbβ3), followed by incubation with an Alexa-633 labeled secondary antibody. Cells positive for both EGFP and DsRed were selected for measuring the PAC-1 binding by flow cytometry. The PAC-1 binding to CHO-αIIbβ3 cells that were transfected with empty vectors was defined as a basal line. Meanwhile, the expressing levels of integrin αIIbβ3 in transfected CHO-αIIbβ3 cells were measured by an antibody for the integrin αIIbβ3 complex, which were further used to normalize the PAC-1 binding.

Integrin α5β1 activation in CHO cells was evaluated by the GST-fused fibronectin type III repeats 9–11 (GST-Fn-III). Briefly, GST-Fn-III was biotinylated and used to incubate with the transfected CHO cells, followed by staining the cells with an Alexa-633 labeled streptavidin. The GST-Fn-III binding to positively transfected cells was quantified by flow cytometry. The basal binding level was defined with the cells that were transfected only with empty vectors. The specificity of GST-Fn-III binding to β1 integrin on CHO cells was verified by employing a CHO cell line lacking integrin α5β1. Meanwhile, the expression levels of endogenous α5β1 in the transfected CHO cells were also measured and used to normalize the GST-Fn-III binding.

In addition, β1-integrin activation was also evaluated in 3 T3 cells by 9EG7 antibody that specifically recognizes the active β1-integrin. 3 T3 cells were transfected with the indicated regulators and used to incubate with 9EG7, followed by incubation with an Alexa-647 labeled secondary antibody. The transfected cells positive for both EGFP and DsRed were selected for measuring the 9EG7 binding by flow cytometry. Binding of 9EG7 to 3 T3 cells transfected with empty vectors was used as a basal line. Meanwhile, the expressing levels of α5β1-integrin in transfected cells were also measured and used for normalizing the 9EG7 binding.

### Cell adhesion and spreading assays

Transfected CHO cells were detached and washed three times with serum-free medium. For cell adhesion assay, harvested cells were used to incubate with coated fibronectin at 37 °C for 15 min; for cell spreading assay, the incubating time increased to 60 min. The wells were washed four times with PBS and the adherent cells were fixed with 4% paraformaldehyde. Finally, the adherent cells were imaged by microscopy and further quantified by Image J software.

### GST pull-down assays

First, GST and GST-tagged integrin CT were incubated with glutathione-Sepharose 4B beads (GE). Then the beads with pre-loaded GST proteins were used to incubate the tested proteins overnight in cold room. After incubation, the beads were extensively washed and proteins bound to the beads were evaluated by SDS-PAGE followed by Coomassie blue staining or immunoblotting. All other GST pull-down experiments described in this study were performed similarly as described above.

### Yeast two-hybrid assays

The Matchmaker™ Gold yeast two-hybrid system was employed according to the manufacturer’s protocol to determine protein-protein interaction (Clontech). Briefly, sharpin and kindlin-1 (or their mutants) were cloned into pGADT7 and pGBKT7 vectors, respectively, for expressing the fusion proteins of AD-sharpin and BD-kindlin-1. Here AD and BD represent the activation domain and the DNA-binding domain of GAL4. Cell clones grown on SD-2 selection media were further plated on SD-4 selection media that lack tryptophan, leucine, histidine and adenine. Histidine and adenine are selection markers for the AD/BD complex formation. Therefore, growth of the transformed yeast cells on SD-4 media indicates the interaction between sharpin and kindlin-1. In this experiment, Bop1 (BLADE-ON-PETIOLE 1) and Bop2 (BLADE-ON-PETIOLE 2), two known binding molecules, were used as positive controls; and empty vectors were used as negative controls.

### siRNA directed knockdown of endogenous sharpin in cells

The siRNA duplex specifically targeting sharpin and one non-targeting siRNA control were synthesized (GenePharma, Shanghai). The siRNA sequences were shown as follows: siRNA1 targeting hamster sharpin: 5′-GCACUGGUACGAGAUGCUATT-3′ (sense strand), and 5′-UAGCAUCUCGUACCAGUGCTT-3′ (antisense strand); siRNA2 targeting hamster sharpin: 5′-GCUCUCAGUGUCCAGCUUATT-3′ (sense strand), and 5′-UAAGCUGGACACUGAGAGCTT-3′ (antisense strand); non-targeting control siRNA: 5′-UUCUCCGAACGUGUCACGUTT-3′ (sense strand), and 5′-ACGUGACACGUUCGGAGAATT-3′ (antisense strand); siRNAa targeting mouse sharpin: 5′-GCGGAAGCUGCAAUUGAUATT-3′ (sense strand), and 5′-UAUCAAUUGCAGCUUCCGCTT-3′(antisense strand); siRNAb targeting mouse sharpin: 5′-GCAUCAUGUGGCUCUCAAUTT-3′ (sense strand), and 5′-AUUGAGAGCCACAUGAUGCTT-3′(antisense strand); siRNAc targeting mouse sharpin: 5′-CCGGAAAUUAGGCUUGUUUTT-3′ (sense strand), and 5′-AAACAAGCCUAAUUUCCGGTT 3′ (antisense strand). Specific siRNA that target sharpin and non-targeting control siRNA were transiently transfected into CHO cells or 3 T3 cells, and their ability to knock down endogenous sharpin was determined by immunoblotting.

### NMR spectroscopy

Two-dimensional HSQC experiments used to examine the interaction between sharpin and the integrin β CT were performed on Bruker 600MHZ spectrometers equipped with a triple resonance probe at 25 °C in 50 mM Tris, 50 mM NaCl, 1 mM DTT, PH 6.7.

### SPR

Real time protein-protein interaction was analyzed using a Biacore8K instrument (GE). Purified sharpin protein was coupled to carboxymethyl dextran of CM5 biosensor chips according to the manufacturer’s instruction. Experiments were performed at room temperature in PBS buffer. SPR sensograms were obtained by injecting various concentrations of analytes (the integrin β1 CT and the integrin β3 CT proteins). The chip surfaces were regenerated by injecting a short pulse of glycine (pH 2.0). The resulting sensograms were analyzed in overlay plots using BIA evaluation software.

### Statistical analysis

Results represent the mean ± SD which are calculated from at least three independent experiments. Statistical significance was calculated using a two-tailed Student’s *t*-test. More than two groups were compared using the One-way ANOVA post hoc test. A value of *P* < 0.05 was considered significant.

## Results

### Sharpin has different effects on regulating integrin α5β1 and integrin αIIbβ3 activation

**Fig. 1:**
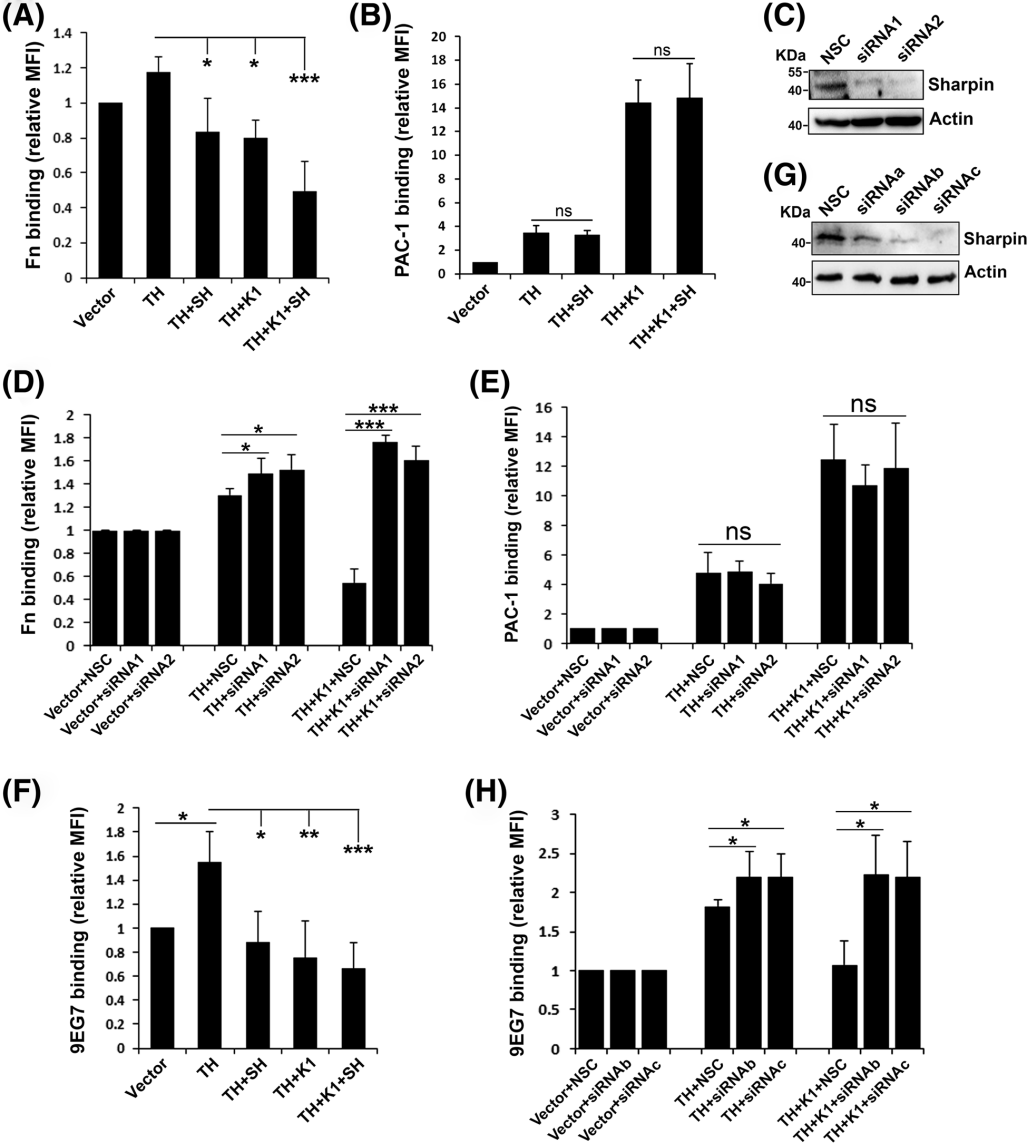
Sharpin suppresses integrin α5β1 activation but not integrin αIIbβ3 activation. **a** Sharpin (SH) was co-expressed in CHO cells together with DsRed-fused talin head (TH) and EGFP-fused kindlin-1 (K1) by transient transfection. Activation of endogenous integrin α5β1 in transfected cells were measured by the GST-Fn-III binding assay. **b** CHO cells that stably express αIIbβ3 (CHO-αIIbβ3) were used to express the indicated regulators. Their effects on integrin αIIbβ3 activation were evaluated by the PAC-1 antibody binding assay. **c** CHO cells were transfected with two different siRNA (siRNA1 and siRNA2) specifically targeting endogenous sharpin in CHO cells or non-targeting siRNA (NCS) as a control. 24 h after transfection, expression of sharpin protein in CHO cells was evaluated by immunoblotting. **d**, **e** NSC or two siRNA targeting hamster sharpin were co-transfected either in CHO cells (**d**) or CHO-αIIbβ3 cells (**e**) together with DsRed-fused talin head (TH) or TH plus EGFP-kindlin-1 (K1); their effects on integrin α5β1 activation in CHO cells or integrin αIIbβ3 activation in CHO-αIIbβ3 cells were evaluated by the GST-Fn-III binding assay and the PAC-1 antibody binding assay, respectively. **f** Sharpin (SH) was co-expressed in 3 T3 cells together with DsRed-fused talin head (TH) and EGFP-fused kindlin-1 (K1) by transient transfection. Activation of endogenous integrin α5β1 in transfected 3 T3 cells was measured by the 9EG7 antibody binding assay. **g** 3 T3 cells were transfected with three different siRNA (siRNAa, siRNAb and siRNAc) targeting endogenous sharpin in 3 T3 cells or non-targeting siRNA (NCS) as a control. 24 h after transfection, expression of endogenous sharpin protein in 3 T3 cells was evaluated by immunoblotting. **h** NSC or two different siRNA (siRNAb and siRNAc) were co-transfected in 3 T3 cells together with DsRed-fused talin head (TH) or TH plus EGFP-kindlin-1 (K1), and their effects on integrin α5β1 activation in 3 T3 cells were evaluated by the 9EG7 antibody binding assay. The results represent the mean ± SD of at least 3 experiments. (MFI: median of fluorescence intensity; ns, not significant; *, *p < 0.05*; **, *p < 0.01*; ***, *p < 0.001*)

Previously it was reported that sharpin could directly bind to a conserved region in the CTs of integrin α1, α2, and α5 subunits and inhibit β1 integrin activation in cancer cells [32]. Since sharpin is widely expressed, it may possibly act as a common inhibitor of activation for different integrin members. To test this idea, we compared the roles of sharpin in regulating integrin α5β1 and integrin αIIbβ3 activation induced by either the talin head domain alone or the talin head domain plus kindlin-1 in CHO cells that express endogenous integrin α5β1 or CHO-αIIbβ3 cells that stably express exogenous integrin αIIbβ3. The talin head domain, kindlin-1 and sharpin that were fused with DsRed, EGFP and flag tags respectively were transiently expressed in CHO or CHO-αIIbβ3 cells. To ensure the expression of the talin head domain and kindlin-1, transfected cells that were positive for both DsRed and EGFP were selected for functional analyses. Meanwhile, the expression of flag-tagged sharpin was validated by immunoblotting. Integrin activation was evaluated by either the GST-Fn-III binding assay for integrin α5β1 or the PAC-1 binding assay for integrin αIIbβ3. As expected, sharpin significantly suppressed the talin head domain mediated β1-integrin activation (Fig. 1a), which verifies its negative role in regulating β1-integrin activation, as previously described [32]. Interestingly, sharpin failed to suppress the talin head domain mediated integrin αIIbβ3 activation (Fig. 1b). These results suggest that the role of sharpin in regulating integrin activation can be integrin specific. Consistent with the finding from a previous study [18], we also verified that kindlin-1 exhibited distinct functions on regulating integrin α5β1 and αIIbβ3 activation. As shown in Fig. 1a and b, kindlin-1 could inhibit talin head domain mediated integrin α5β1 activation while it adversely enhanced talin head domain mediated integrin αIIbβ3 activation. Importantly, sharpin could further suppress integrin α5β1 activation but had no significant effect on integrin αIIbβ3 activation when it was co-expressed with the talin head domain and kindlin-1. It is worth noting that the observed regulation of sharpin and kindlin-1 on integrin α5β1 activation in CHO cells is not due to the alternately expressed levels of surface β1-integrin since they were very similar across different transfectants (data not shown). Also, the ligand binding was also normalized by the levels of surface expressed integrin. Together, these results suggest that sharpin may regulate integrin activation in an integrin-specific manner.

As we measured by qPCR, sharpin is also endogenously expressed in CHO cells (~ 0.5% of actin at mRNA levels). We next evaluated the role of endogenous sharpin in regulating integrin α5β1 and integrin αIIbβ3 activation. As shown in Fig. 1c, the expression of endogenous sharpin in CHO cells was significantly suppressed by two siRNA specifically for hamster sharpin. As expected, knocking down endogenous sharpin in CHO cells significantly enhanced the talin head domain induced integrin α5β1 activation (Fig. 1d). Knockdown of endogenous sharpin could substantially reverse the inhibition of kindlin-1 on integrin α5β1 activation, indicating that kindlin-1 may functionally couple with endogenous sharpin in inhibiting integrin α5β1 activation. However, knockdown of endogenous sharpin had no effect on regulating integrin αIIbβ3 activation mediated by the talin head domain or the talin head plus kindlin-1 (Fig. 1e), further verifying that the inhibition of sharpin on integrin activation is specific for integrin α5β1 but not integrin αIIbβ3.

To further specify the regulation of sharpin on conformational changes of β1-integrin, we employed 9EG7, an antibody that specifically recognizes activated β1-integrin of mouse. Here, we utilized mouse 3 T3 cells. As shown in Fig. 1f, expression of the talin head domain significantly induced 9EG7 binding in 3 T3 cells. Meanwhile, overexpression of sharpin or kindlin-1, or both, significantly reduced the talin head domain induced 9EG7 binding. On the other hand, three siRNA were designed to be used to knock down endogenous sharpin in 3 T3 cells (Fig. 1g). When co-expressing the selected sharpin siRNA with the talin head domain or the talin head domain plus kindlin-1, significantly enhanced 9EG7 binding was observed (Fig. 1h). Collectively, these results suggest that sharpin and kindlin-1 can serve as negative regulators of β1-integrin activation in different cells.

### Sharpin directly interacts with the integrin β1 CT and impedes Talin head domain binding

**Fig. 2:**
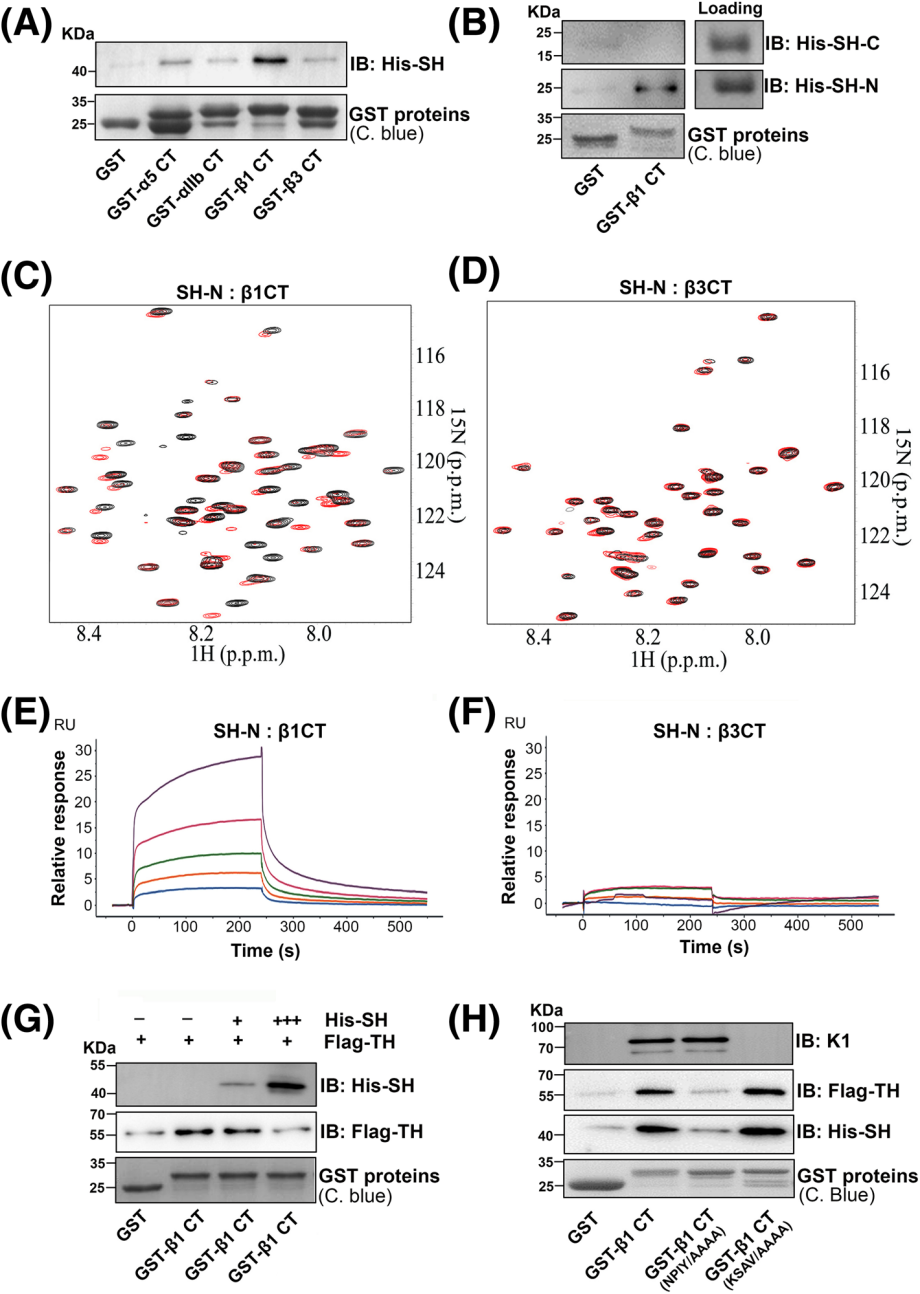
Sharpin directly binds to the integrin β1 CT and inhibits the talin head domain binding. **a** Purified GST and GST-fused integrin CT, as indicated, were coupled to Glutathione Sepharose beads and used to incubate with his-tagged sharpin (His-SH). After incubation, the beads were extensively washed and proteins bound to the beads were eluted by boiling the beads in laemmli sample buffer. GST proteins loaded on the beads and co-precipitated His-SH were evaluated by SDS-PAGE followed by Coomassie blue (C. blue) staining and immunoblotting (IB). **b** The N-terminus (SH-N, 1–217 amino acids) and C-terminus (SH-C, 217–387 amino acids) of sharpin were expressed and purified with a his tag and used to test their binding to GST or GST-β1 CT, as described in (**a**). **c** Selected region of HSQC spectra of 50 μM ^15^N-labeled β1 CT in the absence (black) and presence (red) of 250 μM N-terminus of sharpin (SH-N). **d** Selected region of HSQC spectra of 50 μM ^15^N-labeled β3 CT in the absence (black) and presence (red) of 250 μM N-terminus of sharpin (SH-N). **e**, **f** Purified protein of the N-terminus of sharpin was immobilized on CM5 chip surfaces. Various concentrations (2.5 μM, 5 μM, 10 μM, 20 μM and 40 μM) of either the integrin β1 CT protein (**e**) or the integrin β3 CT protein (**f**) were injected and passed over the chips, and the binding curves were recorded on a Biacore 8 K instrument. **g** Purified GST and GST-β1 CT proteins were loaded onto Glutathione Sepharose beads which were then used for incubating with flag-fused talin head (Flag-TH) in the presence or absence of his-sharpin (His-SH) with different ratios. After incubation, the beads were washed and co-precipitated proteins were measured by SDS-PAGE followed by Coomassie blue (C. blue) staining and immunoblotting (IB). **h** Purified GST, GST-β1 CT and GST-β1 CT mutants that carry the NPIY/AAAA mutations or the KSAV/AAAA mutations were coupled to Glutathione Sepharose beads and used to incubate with His-SH, Flag-TH or kindlin-1 (K1) proteins, respectively. Binding of His-SH, Flag-TH or K1 to these GST proteins were evaluated by SDS-PAGE followed by immunoblotting (IB). Meanwhile, the loaded GST proteins on the beads were also measured by Coomassie blue (C. blue) staining

The effect of specific inhibition of sharpin on integrin α5β1 activation but not integrin αIIbβ3 activation provoked us to explore the possible mechanisms. Since sharpin was previously found to interact with the integrin α CT, we then tested the ability of sharpin bind to the CTs of both integrin α5β1 and integrin αIIbβ3. GST and GST-fused CT proteins of the integrin α5, β1, αIIb and β3 subunits were loaded on Glutathione Sepharose beads and incubated with his-tagged sharpin. After incubation, the beads were extensively washed and sharpin bound to the GST-fused integrin CTs was evaluated by immunoblotting. As shown in Fig. 2a, the integrin β1 CT was able to interact with sharpin. The integrin α5 CT also interacted with sharpin but the binding signal was moderate. Comparatively, the CTs of integrin αIIb and β3 only exhibited minimal binding to sharpin. In addition, we found that the N-terminal region (1–217) but not the C-terminal region (217–387) of sharpin was able to interact with the integrin β1 CT (Fig. 2b). Next, an NMR approach was employed to verify the interaction of sharpin with the integrin β1 and β3 CTs. As shown in Fig. 2c and d, when the N-terminal fragment of sharpin was added to the ^15^N-labeled integrin β1 CT, significant chemical shift changes occurred, suggesting that these two proteins interact with each other. However, significant chemical shift changes did not appear when the sharpin fragment was added to the ^15^N-labeled integrin β3 CT. Meanwhile, we also performed SPR experiments by injection of the integrin β1 CT protein or the integrin β3 CT protein and then allowing them to flow over immobilized N-terminal fragment of sharpin. We found that the integrin β1 CT, but not the integrin β3 CT, displayed binding curves by which the binding affinity was calculated at a *K*_*d*_ of 40 × 10^− 6^ M (Fig. 2e and f). Together, these results demonstrate that sharpin can substantially interact with the integrin β1 CT while its interaction with the integrin β3 CT is relatively mild. The preference of sharpin binding to the integrin β1 CT may define its functional specificity on inhibiting integrin α5β1.

Because sharpin can interact with the integrin β1 CT and also suppress talin head domain mediated β1-integrin activation, we next tested by pull-down assays if sharpin could affect the talin head domain binding to the integrin β1 CT. As shown in Fig. 2g, sharpin not only interacted with the integrin β1 CT, but also inhibited talin head domain binding to the integrin β1 CT in a dose dependent manner, indicating that sharpin can compete with the talin head domain to bind the integrin β1 CT. As known, the talin head domain interacts with the membrane-proximal NPIY motif in the integrin β1 CT [7, 12, 39, 40]. When the NPIY motif was substituted with AAAA, the mutated integrin β1 CT did not interact with the talin head domain (Fig. 2h), as expected. Importantly, this β1 CT mutant no longer interacted with sharpin either, suggesting that the binding sites of sharpin and the talin head domain in the integrin β1 CT overlap. As a control, the NPIY/AAAA mutations in the integrin β1 CT has no effect on kindlin-1 binding. When the KSAV residues next to the NPIY motif were mutated to AAAA in the integrin β1 CT, the mutated integrin β1 CT still interacted with the talin head domain or sharpin, but failed to interact with kindlin-1 (Fig. 2h). Together, these results show that sharpin may competitively block the talin head domain binding to the integrin β1 CT, by which it suppresses β1-integrin activation.

### Kindlin-1 interacts with sharpin and facilitates recruitment of sharpin to the integrin β1 CT

**Fig. 3:**
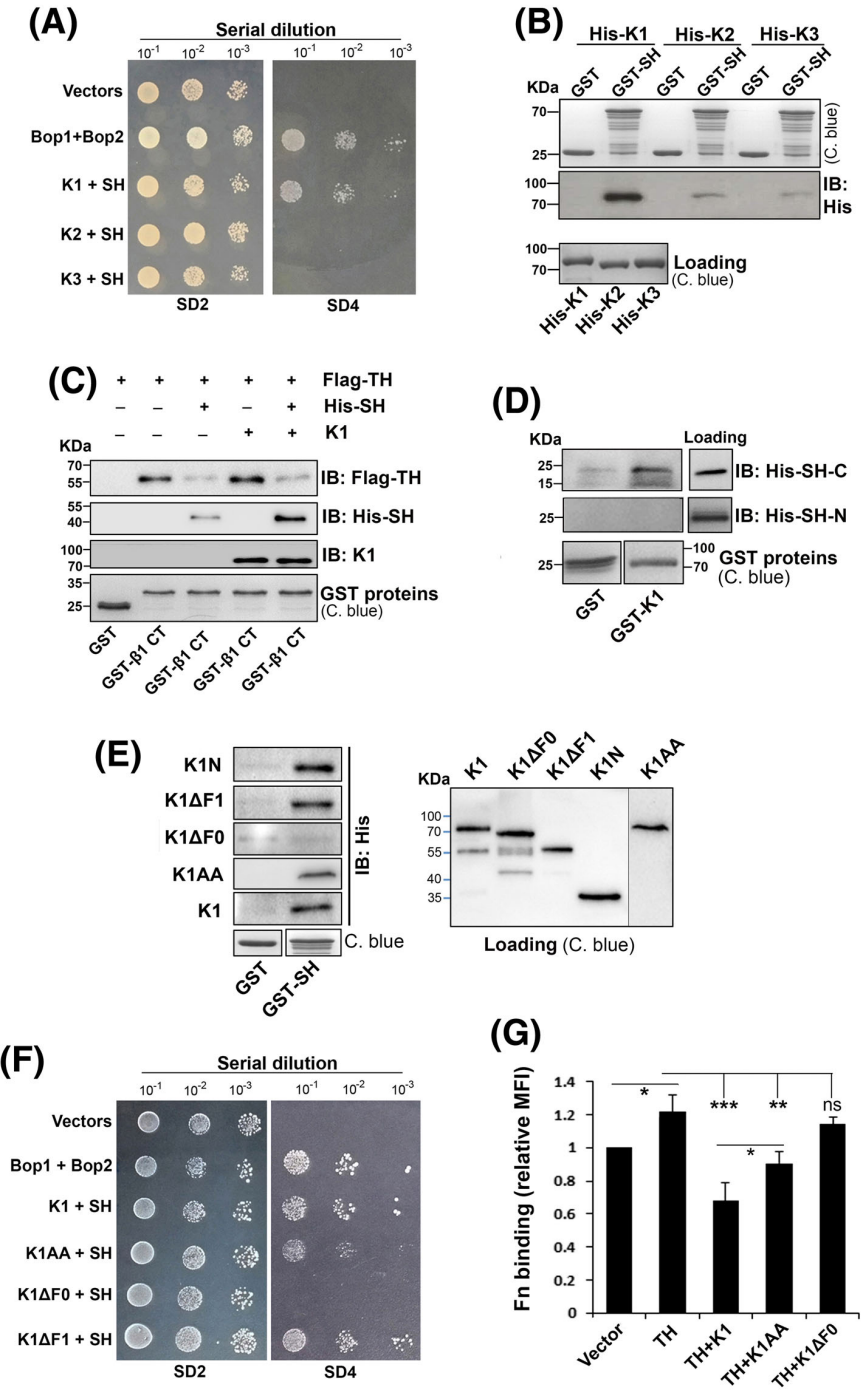
Kindlin-1 directly interacts with sharpin (via its F0 subdomain) and recruits sharpin to inhibit β1-integrin activation. **a** The kindlin family members (K1, K2 and K3) were fused with the DNA-binding domain of Gal4 in pGBKT7 vector and sharpin (SH) was fused with the transcriptional activation domain of Gal4 in pGADT7 vector. The interaction between kindlin and sharpin was evaluated using the Matchmaker™ Gold yeast two-hybrid system by a serial dilution method on selection media. Two molecules known for interacting with each other (Bop1/Bop2) were used as a positive control and the empty vectors were used as a negative control. Growth of yeast cells on SD2 selection media indicates successful transformation; growth of yeast cells on SD4 selection media indicates a positive protein-protein interaction. **b** Glutathione Sepharose beads were loaded with GST and GST-fused sharpin (GST-SH) proteins and used to incubate with his-tagged kindlins (His-K1, His-K2 and His-K3). The loading of GST and GST-SH on the beads was measured by Coomassie blue (C. blue) staining. Binding of His-kindlins to GST proteins was analyzed by immunoblotting (IB). **c** Purified GST and GST-β1CT proteins were coupled to Glutathione Sepharose and used to incubate with flag-tagged talin head (Flag-TH) in the presence or absence of his-tagged sharpin (His-SH) and/or kindlin-1 (K1). After incubation, beads were extensively washed. The loading of GST proteins was evaluated by Coomassie blue (C. blue) staining. Precipitated protein samples on the beads, including Flag-TH, His-SH and K1, were evaluated by SDS-PAGE followed by immunoblotting (IB). **d** Glutathione Sepharose beads were loaded with GST and GST-fused kindlin-1 (GST-K1) proteins and used to incubate with his-tagged N-terminus (His-SH-N) or C-terminus (His-SH-C) of sharpin. The loading of GST and GST-K1 on the beads was measured by Coomassie blue (C. blue) staining. Binding of His-SH-N or His-SH-C to GST proteins was analyzed by immunoblotting (IB). **e** Kindlin-1 (K1) and its mutants, including K1ΔF0, K1ΔF1, the N-terminal fragment (F0 + F1) of kindlin-1 (K1N), and the kindlin-1 QW/AA mutant (K1AA), were expressed and purified with a his tag. Interaction of GST or GST-fused sharpin (GST-SH) with these kindlin-1 proteins were evaluated by pull-down assays followed by immunoblotting (IB). **f** Interaction of sharpin with kindlin-1 and its mutants were also evaluated using the Matchmaker™ Gold yeast two-hybrid system, same as described in (**a**). **g** The effects of kindlin-1 mutants, including K1AA and K1ΔF0, on integrin α5β1 activation in CHO cells were evaluated by co-transfection and the GST-Fn-III binding assay. The results represent the mean ± SD of at least 3 experiments. (MFI: median of fluorescence intensity; ns, not significant; *, *p < 0.05*; **, *p < 0.01*; ***, *p < 0.001*)

As demonstrated, kindlin-1 also can inhibit β1-integrin activation (Fig. 1). Interestingly, such an inhibition can be reversed by knocking down endogenous sharpin. The functional correlation between kindlin-1 and sharpin prompted us to test if they can interact with each other. We first employed the yeast two-hybrid system to measure their interaction. As shown in Fig. 3a, the yeast cells transformed with sharpin and kindlin-1 that were respectively fused with the activation domain and the DNA-binding domain of GAL4 were able to grow on selection media, suggesting they interact with each other in yeast cells. However, the interaction of sharpin with kindlin-2 and kindlin-3 was not detectable. Further, we performed pull-down assays and observed a similar binding pattern between sharpin and the kindlin family members, in which sharpin significantly interacted with kindlin-1 while its interaction with kindlin-2 and kindlin-3 were minimal (Fig. 3b). These results suggest that sharpin prefers to interact with kindlin-1 among the kindlin family members.

Identification of the direct interaction between sharpin and kindlin-1 drove us to test if kindlin-1 can facilitate recruitment of sharpin to the integrin β1 CT. As shown in Fig. 3c, in the presence of kindlin-1, the association of sharpin with the integrin β1 CT significantly increased, and concomitantly, the talin head domain binding to the integrin β1 CT dramatically decreased. Importantly, we found that the binding sites of kindlin-1 and the integrin β1 CT in sharpin were different. Kindlin-1 interacted with the C-terminal fragment of sharpin while the integrin β1 CT interacted with the N-terminal fragment of sharpin (Figs. 2b and 3d). Therefore, the mutual interaction of the integrin β1 CT, kindlin-1 and sharpin may enhance the blockage of the talin head domain binding to the integrin β1 CT, by which sharpin and kindlin-1 inhibit β1-integrin activation.

### Kindlin-1 interacts with sharpin via its F0 subdomain

Next, we wanted to identify the subdomain in kindlin-1 that is responsible for sharpin binding. Based on a previous test using the yeast two-hybrid system, we found that the N-terminal fragment of kindlin-1 that consists of the F0 and F1 subdomains was able to interact with sharpin (data not shown). Therefore we focused on this N-terminal fragment of kindlin-1 and verified its interaction with sharpin in the pull-down experiments (Fig. 3e). In addition, we found that kindlin-1 with a deletion of the F0 subdomain failed to interact with sharpin while kindlin-1 missing the F1 subdomain still interacted with sharpin (Fig. 3e), suggesting that the F0 subdomain in kindlin-1 is involved in the interaction with sharpin. Moreover, we found that the kindlin-1 QW/AA mutant that is defective for binding to integrin was still able to interact with sharpin, showing that the binding sites of sharpin and the integrin β1 CT in kindlin-1 are distinct. Moreover, we also verified these binding results using the yeast two-hybrid assay (Fig. 3f). These observations further support the mutual interaction of these three proteins.

In line with the binding results, kindlin-1 with a deletion of the F0 subdomain no longer inhibited the talin head domain mediated β1-integrin activation (Fig. 3g). Although the kindlin-1 QW/AA mutant still had an inhibitory effect on β1-integrin activation, such an inhibition was significantly compromised when compared to wild type kindlin-1 (Fig. 3g), indicating that the inhibition of kindlin-1 on β1-integrin activation relies, at least partially, on its interaction with the integrin β1 CT. Collectively, these results suggest that the inhibition of kindlin-1 on β1-integrin activation may be implemented by facilitating recruitment of sharpin, and subsequently restricting the talin head domain binding to the integrin β1 CT.

### Kindlin-1 suppresses β1-integrin mediated cell adhesion and signaling

**Fig. 4:**
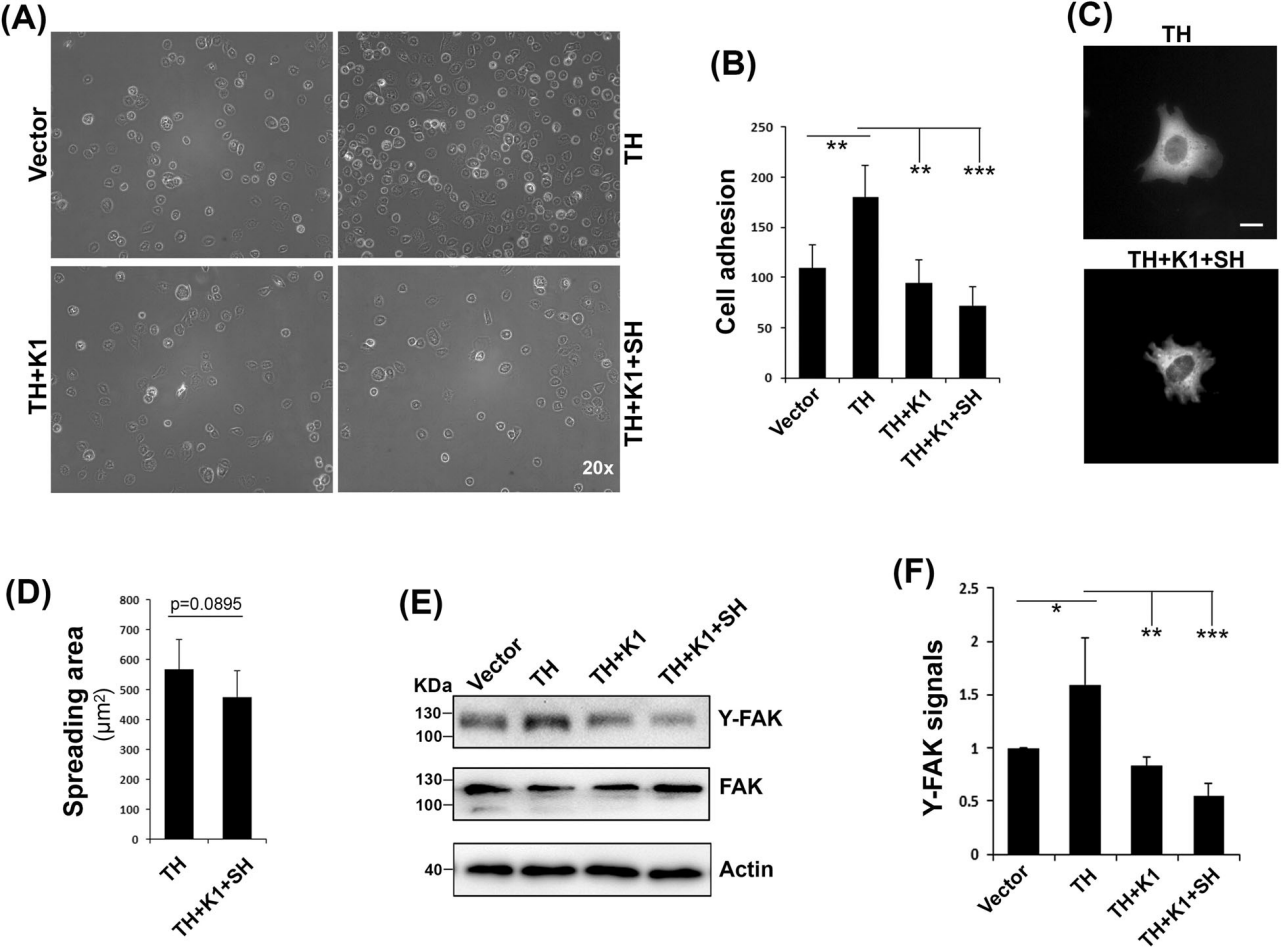
Kindlin-1 suppresses β1-integrin-mediated cell adhesion and signaling. (A & B) CHO cells expressing the indicated regulators were used to incubate with coated fibronectin for 15 min. After washing and fixation, adherent cells were imaged (**a**) and counted (**b**). **c** CHO cells expressing the talin head (TH) plus empty vectors and TH plus kindlin-1 (K1) and sharpin (SH) were allowed to spread on coated fibronectin for 60 min and then fixed. Representative images of spreading cells were shown. Bar distance was 10 μm. **d** Spreading areas of cells were calculated with ImageJ software. **e** Adherent cells on fibronectin were directly lysed for immunoblotting using antibodies for focal adhesion kinase (FAK), tyrosine-phosphorylated FAK (Y-FAK) and actin. **f** The relative density of Y-FAK signals, as shown in (**e**), were quantified using ImageJ software. The results represent the mean ± SD of at least 3 experiments. (*, *p < 0.05*; **, *p < 0.01*; ***, *p < 0.001*)

Based on the above findings that kindlin-1 can suppress β1-integrin activation by recruiting sharpin, we next sought to see if kindlin-1 together sharpin can also affect β1-integrin mediated cell adhesion and spreading. As shown in Fig. 4a and b, overexpression of the talin head domain in CHO cells promoted cell adhesion on immobilized fibronectin; when kindlin-1 was co-expressed, cell adhesion was significantly suppressed; and co-expression of kindlin-1 together with sharpin further diminished cell adhesion. However, late stage cell spreading was only slightly suppressed when kindlin-1 and sharpin were co-expressed with the talin head (Fig. 4c and d). Together, these results demonstrate that kindlin-1 and sharpin significantly affect β1-integrin mediated cell adhesion. Consistently, expression of kindlin-1 or kindlin-1 and sharpin together also reduced FAK activation upon adhesion (Fig. 4e and f). Therefore, these findings show that the kindlin-1-sharpin axis can negatively affect both inside-out and outside-in signaling of β1-integrin.

## Discussion

**Fig. 5:**
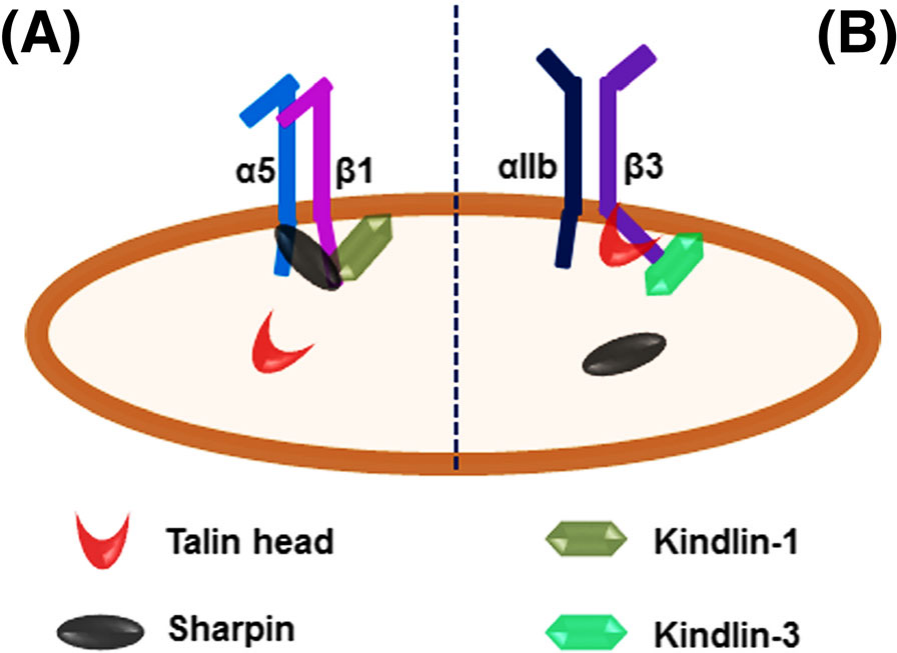
A model to show the different roles of sharpin in regulating integrin α5β1 and αIIbβ3 activation. **a** Sharpin directly interacts with the integrin β1 CT and kindlin-1 as well, thus being able to competitively inhibit the talin head binding to the β1 CT and leading to a negative regulation on integrin α5β1 activation. **b** Sharpin fails to interact with the integrin β3 CT, which is unlikely to affect the talin head binding to the β3 CT. In addition, kindlin-3, the dominant kindlin member in platelets to support integrin αIIbβ3 activation, also exhibits significantly compromised binding to sharpin

Sharpin, one key component of the linear ubiquitin assembly complex, is involved in regulating NF-κB activation in innate immunity [41]. Interestingly, a previous study showed that sharpin could inhibit β1-integrin activation by directly interacting with the CTs of the integrin β1-associated α subunits (such as α1, α2 and α5) at a conserved membrane-proximal region, and this interaction could disrupt recruitment of talin and kindlin, two key integrin activators, to the integrin β1 CT [32]. In addition, a study from the same group disclosed that sharpin could also interact with the integrin αL CT and inhibit integrin LFA-1 activation [36]. These studies suggest that sharpin may serve as a common inhibitor for different integrin members since the integrin α CTs share a highly conserved membrane-proximal region. However, in this study, we surprisingly reveal that sharpin fails to inhibit integrin αIIbβ3 activation in CHO-αIIbβ3 cells while it does inhibit β1-integrin activation in CHO cells and 3 T3 cells (Fig. 1). Importantly, we detect that sharpin can interact with the integrin β1 CT (Fig. 2), which was not observed in the previous study [32]. Comparatively, the interaction of sharpin with the CTs of both the integrin αIIb and β3 subunits is considerably moderate (Fig. 2a). Hence, the preference of sharpin to bind the integrin β1 CT but not the β3 CT is in line with its functional specificity in inhibiting integrin α5β1 activation but not integrin αIIbβ3 activation (Fig. 5).

Nonetheless, the molecular basis of the binding specificity of sharpin to the β1 CT but not the β3 CT remains to be determined. As demonstrated, the first NPxY motif in the β1 CT, which is a known binding site for the talin head domain [7, 42], is also involved in interacting with sharpin (Fig. 2h). Since this NPxY motif is highly conserved across different integrin β CTs, it is conceivable that some nonconserved residues in the β1 CT should participate in the binding and possibly determine the binding specificity. As expected, sharpin can inhibit the talin head domain binding to the integrin β1 CT (Fig. 2g), which highlights a novel mechanism by which sharpin is able to suppress β1-integrin activation. In addition, sharpin also has the ability to interact with the integrin α5 CT (Fig. 2a). Because there is no homology between the β1 and the α5 CTs, we speculate that sharpin may interact with these two integrin CTs through different subdomains. Currently, it is unknown whether sharpin can simultaneously interact with these two integrin CTs. Theoretically, possession of two binding sites in the integrin α5β1 CTs for sharpin may facilitate sharpin’s recruitment, and thus facilitate its inhibition on the talin head binding to the β1 CT. To further delineate the functional specificity, the key residues in the integrin β1 CT involved in sharpin binding need to be identified and evaluated in future.

Another novel finding in this study is that sharpin can interact with kindlin-1 (Fig. 3a and b). Interestingly, sharpin preferentially interacts with kindlin-1 but not kindlin-2 or kindlin-3. Because there is no significant interaction between the talin head domain and kindlin [17], kindlin-1, as an integrin binding partner, may recruit sharpin but not the talin head to integrin (Fig. 3c), which can facilitate sharpin binding to the integrin β1 CT and subsequently inhibit the talin head domain binding to the β1 CT in a competitive manner. In fact, kindlin-1 does inhibit the talin head mediated β1-integrin activation (Fig. 1). Since sharpin only minimally interacts with the integrin β3 CT (Fig. 2a, d and f) and has no significant effect on integrin αIIbβ3 activation in CHO-αIIbβ3 cells (Fig. 1b), it seems that the binding capacity of sharpin to the integrin β CT determines its functional consequence on different integrin members. Nonetheless, the kindlin-1 mutant defective for binding to the integrin β1 CT still partially suppresses β1-integrin activation (Fig. 3g), and so does kindlin-2 as demonstrated in another study [18], suggesting that the inhibition of kindlin-1 on β1-integrin activation may also involve additional mechanisms.

Based on our findings, we propose that sharpin may play different roles in regulating integrin α5β1 and αIIbβ3 activation in cells (Fig. 5). Sharpin can be recruited by kindlin-1 and bind to the integrin β1 CT, which is able to block the talin head domain binding to the β1 CT, thus inhibiting integrin α5β1 activation. Here, kindlin-1 and sharpin cooperatively work with each other in inhibiting integrin α5β1 activation. However, due to the weak interaction between sharpin and the integrin αIIbβ3 CTs, the capacity of sharpin to block the talin head domain binding to the integrin β3 CT can be significantly compromised, which may lead it to be less effective on inhibition of integrin αIIbβ3 activation. Presumably, the expressing levels and the availability of sharpin in different cells may eventually define its role in regulating activation for different integrin members. Interestingly, in a recently published paper, the authors observed that knockdown of endogenous sharpin in human iPS cells led to enhanced fibrinogen binding to differentiated megakaryocytes and platelets [43], suggesting that sharpin actually may be able to inhibit integrin αIIbβ3 activation. Nonetheless, due to the relatively weak interaction of sharpin with the integrin αIIbβ3 CTs as well as kindlin-3, the dominant kindlin member in platelets, a significant inhibition of sharpin on integrin αIIbβ3 activation in platelets through blocking the talin head domain binding to the β3 CT is not expected. However, sharpin may participate to inhibit β1-integrin activation in platelets by directly binding to the integrin β1 CT, which possibly contributes to the observed phenotypes. In addition, other unknown mechanisms might also be involved. Therefore, further studies are required to delineate the role of sharpin in regulating β1-integrin activation and β3-integrin activation in platelets and other cells.

## Conclusion

Our study demonstrates that sharpin can directly interact with the integrin β1 CT and kindlin-1 and suppress β1-integrin activation, revealing a novel mechanism in regulating integrin signaling. However, the exact role of sharpin in regulating integrin activation in different cells can be fine-tuned by the stoichiometry/affinity between the participated binding partners.

## Data Availability

Please contact the corresponding authors for all data requests.

## Abbreviations

CT: Cytoplasmic tail
NMR: Nuclear Magnetic Resonance
SPR: Surface Plasmon Resonance
